# The Community Health Systems Reform Cycle: Strengthening the Integration of Community Health Worker Programs Through an Institutional Reform Perspective

**DOI:** 10.9745/GHSP-D-20-00429

**Published:** 2021-03-15

**Authors:** Nan Chen, Mallika Raghavan, Joshua Albert, Abigail McDaniel, Lilian Otiso, Richard Kintu, Melissa West, David Jacobstein

**Affiliations:** a Last Mile Health, Washington, DC, USA.; b LVCT Health, Nairobi, Kenya.; c The Palladium Group, Kampala Uganda.; d VillageReach, Seattle, WA, USA.; e U.S. Agency for International Development, Washington, DC, USA.

## Abstract

To develop guidance for governments and partners seeking to scale community health worker programs, we developed a conceptual framework, collected observations from the scale-up efforts of 7 countries, workshopped the framework with technical groups and with country stakeholders, and reviewed literature in the areas of health and policy reform, change management, institutional development, health systems, and advocacy. We observed that successful scale-up is a complex process of institutional reform. Successful scale-up: (1) depends on a carefully choreographed, problem-driven political process; (2) requires that scaled program models are drawn from solutions that are available in a given health system context and aligned with the resources, capabilities, and commitments of key health sector stakeholders; and (3) emerges from iterative cycles of learning and improvement, rather than a single, linear scale-up effort. We identify stages of the reform process associated with each of these 3 findings: problem prioritization, coalition building, solution gathering, design, program readiness, launch, governance, and management and learning. The resulting Community Health Systems Reform Cycle can be used by government, donors, and nongovernmental partners to prioritize and design community health worker scale-up efforts, diagnose challenges or gaps in successful scale-up and integration, and coordinate the contributions of diverse stakeholders.

## Abstract (precis)

Efforts to scale community health worker programs within primary health care systems in 7 countries illustrated that these efforts are best understood as a complex process of institutional reform. Successful scale up depends on a problem-driven political process; requires that models develop solutions that align with resources, capabilities, and commitments of key stakeholders; and emerges from iterative cycles of learning and improvement.

## Teaser

**Key Findings:**

- Despite strong interest and policy commitments from many countries, models for community-based primary health service delivery have been slow to achieve full scale or deliver quality that matches their potential.
- Reform requires a carefully choreographed effort to enroll key stakeholders to support change by spotlighting a locally relevant gap in the existing health system's performance.
- Designing and launching community health programs depends heavily on local context and must draw from models that are available within a given health system and that align with the resources, capabilities, and commitments of key stakeholders.
- Reform should be viewed as an ongoing adaptive and evolutionary process; learning, governance, and management systems must be designed with this trajectory in mind.

**Key Implications:**

Ministries of health, development partners, and other reform stakeholders can:

- Use the principles in the reform cycle process to identify gaps and priorities for technical work or investment
- Examine community health efforts to identify where technical approaches can be complemented by political, institutional, and reform approaches
- Critically examine projects that implement term-limited, externally driven interventions and adapt designs to promote sustained, well-integrated community health programs at scale

## BACKGROUND

The world today faces a daunting global health crisis; despite decades of medical and technological progress, half the world's population^1^ remains without access to primary health care (PHC) services.

Community health workers (CHWs) are essential to realizing strong PHC that is^2^:

*accessible, equitable, safe, of high quality, comprehensive, efficient, acceptable, available and affordable, and will deliver continuous, integrated services that are people-centred and gender-sensitive.*

CHWs can extend access to health services, save lives,^3^ and generate strong returns on investment.^4^ This evidence has culminated in technical guidance such as the 2018 World Health Organization guidelines on CHW programs.^5^ However, many countries that have made policy commitments to scale CHW programs remain stuck in implementation challenges or have fallen short on their targets.^6^ In multiple cases, countries have scaled community health programs only to find that those programs had little effect on access to PHC services or health outcomes such as mortality.^7^^–^^9^ These challenges range from implementation fidelity, governance, management, and financial resources. For those countries that have achieved success it is often not understood or documented how success has been achieved.^10^ Recent studies on “exemplars” in community health have started to unpack this “black box.”^11^

Recent studies on “exemplars” in community health have started unpacking the “black box” on falling short or achieving success.

In response to this challenge, the U.S. Agency for International Development (USAID), United Nations Children's Fund (UNICEF), and the Bill and Melinda Gates Foundation formed the Integrating Community Health Program (ICH), a collaboration^12^ to advance community-based service-delivery models in 7 countries: Bangladesh, Democratic Republic of the Congo, Haiti, Kenya, Liberia, Mali, and Uganda. ICH-supported partner organizations in each of these countries have worked with their respective ministries of health to scale, strengthen, or sustain community health programs.

As part of this collaboration, we present a framework for community health reform, draw on lessons learned from across the 7 countries' institutionalization efforts, and provide guidance on CHW institutionalization within government-managed health systems (The Supplement contains more details on the framework development). This article explores how an institutional reform perspective may guide practitioners in forging through the persistent implementation failures that have been experienced by governments seeking to scale CHW programs (Box).^13^^–^^15^

We explore how an institutional reform perspective may guide practitioners in forging through persistent implementation failures.

BOX **Defining Institutionalization and Integration in the Context of Community Health Reform**

For the purposes of this discussion, we define “institutions” as the formal and informal norms that structure political, economic, and social interactions.^14^ In the context of community health systems, these include formal norms like policies, program designs, local laws, government organograms, donor protocols, monitoring frameworks, and informal norms like the beliefs, culture, and practices of communities, nongovernmental organizations, governments, and donors. We define “institutionalization” as the process by which new norms (including effective community health interventions) are identified, introduced, refined, and become the dominant norms within a health system.^15^ We also refer to “integration,” which is a key part of this process whereby aspects of community health programming are adopted into the formal public, primary health care system and coordinate across that system. We note that while this article focuses on institutionalization and integration at a country level, this concept has parallels in the global health policy space as well (for example, the Universal Health Coverage Global Action Plan and its associated “accelerators” for primary health care and health financing can more effectively integrate the community sector). The reform cycle can inform designing global agendas to support national reform.

We summarize 3 key findings from country experiences and the literature:

1. Successful institutionalization efforts depend on a carefully choreographed, problem-driven political process.
2. Successful community-based program models must be drawn from solutions that are available in a given health system context and aligned with the resources, capabilities, and commitments of key health sector stakeholders.
3. Progress toward goals of scale, integration, and quality is the product of iterative cycles of learning and improvement, rather than a single, linear scale-up effort.

We identify and describe the critical stages of this process—the Community Health Systems Reform Cycle. Additional information on this process is available in a Supplement. We draw short vignettes from the 7 ICH partner countries that have navigated through these stages.

## COMMUNITY HEALTH INSTITUTIONALIZATION AS A “REFORM CYCLE”

The Community Health Systems Reform Cycle is illustrated (Figure). Key features that a country may take on at each stage of the reform cycle are listed (Table). The context surrounding reform will differ by country. However, community health reformers generally include government stakeholders, technical and NGO partners, institutional partners, funders, and frontline health workers. These reformers often collaborate through groups or coalitions established that support the reform process including technical working groups, steering committees, or ongoing stakeholder coordination mechanisms.

**TABLE.** Key Country Actions by Community Health Systems Reform Cycle Stage

**Problem Prioritization**	Actors diagnose and frame a compelling problem or opportunity that sets the foundation for the rest of the cycle. A meaningful and relevant problem has been identified, pain points and unmet needs have been defined and, where possible, these are connected to priority areas for reform. Relevant actors acknowledge the need for reform within the community health system, while committing towards a joint vision for addressing gaps.
**Coalition Building**	A group is formed around a compelling problem or vision. Members understand the group and individual roles and goals. Group size and composition is fit for purpose. Diverse membership is established that can fill critical roles for reform (e.g., leaders, connectors, gatekeepers, donors, enablers, change champions, and links to key players outside the coalition).
**Solution Gathering**	Criteria or priorities are developed to determine how to assess solutions. Potential solutions are gathered, drawing from existing, local, and international ideas and where possible, specific ideas for reform are tested/piloted for effectiveness. Promising solutions are prioritized for integration into the health system.
**Design**	Key decision makers, contributors, and authorizers of the reform are identified. These may be a small group, or large multi-sectoral group. Key informants and designers map and understand the different design choices. Where possible, evidence about the different design options and expected cost, impact, and feasibility are identified. Through consultations, workshops, and other forums, groups recommend design choices and decision makers are able to validate these choices. This stage includes the design of training materials, operational plans, job descriptions, management tools, data collection systems, supply chain processes, and planning documents which are all necessary for planning.
**Readiness**	Coalition actors and champions generate buy-in from actors who will need to play key roles in the launch, rollout, and maintenance of the program, including sharing of key information and knowledge. Stakeholders also translate program design into costed operational plans and implementation guidance. These plans should include a clear “launch” plan, accompanied by strong planning and management tools to ensure smooth rollout. Orienting and resourcing stakeholders to fulfill new roles and responsibilities is key. Costed plans inform financing mechanisms to ensure that needed funds are mobilized and can readily flow to the right actors for implementation. Additionally, investment plans for sustainable financing are put in place. Stakeholders identify and address policy/protocol conflicts and integration needs across the health system.
**Launch**	Key actors are able to access relevant skills, knowledge, and resources to execute their new roles. New processes and organizational structures are identified, socialized, and then implemented. As these shifts progress, the program reform is implemented in target areas. From implementation, learning is gathered to demonstrate momentum and identify challenges to achieving scale. Particular attention should be paid to roll out challenges to make shifts in design quickly.
**Governance**	During this stage, stakeholders establish a project governance framework, which includes key leadership and decision-making bodies, clear roles and responsibilities, and explicit decision rights. It is also critical to establish processes for risk and issue management; stakeholder engagement; and cross-functional communication. As the program evolves, actors monitor and assess progress to advance clear decision making and address critical issues or challenges.
**Management and Learning**	Key stakeholders regularly review data to inform joint problem solving (e.g., regular program reviews at national and subnational level). These reviews serve to identify and institutionalize reflection points. Continuous improvement within existing program design is a key feature of this stage, in which challenges and changes to program design and other systems bottlenecks are identified.

The following sections will dive deeper into the specific stages and highlight examples from each country's reform journey, both to demonstrate the features of each stage within the country context and to elevate examples of countries' learning and success.

### Building Political Will: Problem Prioritization and Coalition Building

Our first finding from observing community health scale-up efforts is that the fundamental challenge faced by countries seeking to institutionalize CHW programs is political rather than technical. Successfully achieving scaled and institutionalized CHW programs requires the coordinated support of a range of governmental and nongovernmental stakeholders. The failure to align these stakeholders around a common vision of reform and secure their material support has stalled CHW scale-up efforts in numerous countries, as evidenced by the large number of countries that have formally published CHW scale-up plans but have struggled to secure critical inputs, such as financing, standardized training, or a pathway to absorption of CHWs into the government health system workforce.^16^

The fundamental challenge that countries face when seeking to institutionalize CHW programs is political rather than technical.

This challenge is a familiar one to students of institutional reform processes. Analyses of successful institutional development find that reform depends on critical “authorizing” stakeholders—which may include senior government authorities but also include informal authorities and middle-level and frontline workers—being convinced of the need for institutional change and recruited to participate in defining and implementing the appropriate reform.^13^ This process of institutionalization depends on would-be reformers illustrating the compelling gap in the services provided by the existing system and using that problem to recruit the authorizers needed to mount a large-scale reform effort.

Thus, the Community Health Systems Reform Cycle begins with 2 stages that reflect this essential political process: problem prioritization and coalition building.

#### Problem Prioritization

During the stage of problem prioritization, local reform actors diagnose and frame a compelling problem or opportunity that convinces critical stakeholders of the need for action. Compelling problems harness windows of opportunity, which might include political or economic shocks, routine changes like transitions in administrations, or newly publicized facts (e.g., health statistics), which reformers can frame as urgent provocations in response to which a problem must be prioritized.^17^^,^^18^ To effectively recruit participants into a reform effort, the prioritized problem should be defined by influential local actors within the health system and framed with reference to the dimensions of the problem considered most important by key stakeholders within the system. Examples from Liberia and Haiti illustrate problem prioritization efforts.

**Liberia Prioritized the 2014 Ebola Virus Disease Response for CHW Reform.** Liberia's response to the 2014 Ebola outbreak demonstrates the potential impact of problem prioritization. Key reformers, including President Ellen Johnson Sirleaf, harnessed the focusing power of the epidemic and the dramatic need for new capacity to deliver services at the community level to push forward specific reforms in health. President Sirleaf directly connected the emergency response to CHW reform in a 2014 briefing^19^:

*We are going to make the final push to fight Ebola now, by supporting community workers to get the job done.*

Previous attempts at community health reform faced opposition. For example, many clinicians resisted task shifting the provision of key health services, such as family planning or management of childhood illnesses, to community health volunteers. This, along with other contributing factors, undermined efforts to promote the professionalization and payment of community health volunteers. However, the Ebola epidemic created a window that allowed reformers to both demonstrate the capacity of CHWs to achieve dramatic results and intensify focus on the problem that remote and rural communities were out of reach of essential health services, including Ebola screening, isolation, and referral. The urgency of this challenge attracted a broad coalition of high-level political champions, technical actors, implementers, and donors that were motivated to play a role in the reform. Ultimately, this exploitation of a well-framed problem led to a 2016 revised National Community Health Policy and Strategy that created a new cadre of CHWs, the community health assistants.

**Figure:**
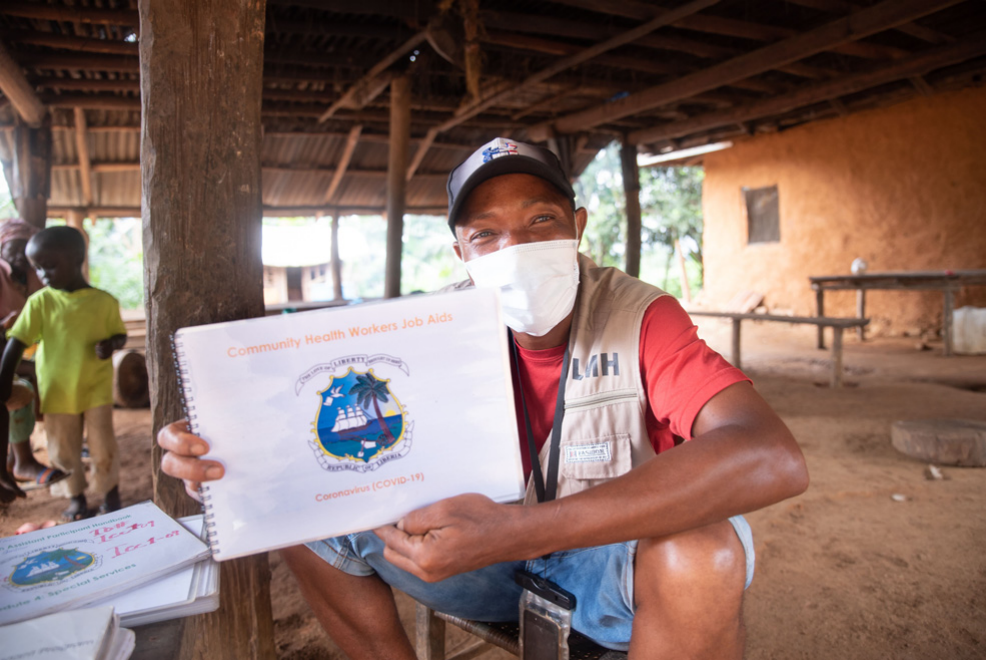
Jerome Gardiner, a community health assistant in Liberia, displays his COVID-19 job aid. Liberia's community health assistants are a cadre of government-formalized community health workers that are paid, supervised, trained, equipped, and integrated into the public health system. © 2020 Rachel Larson/Last Mile Health.

**FIGURE:**
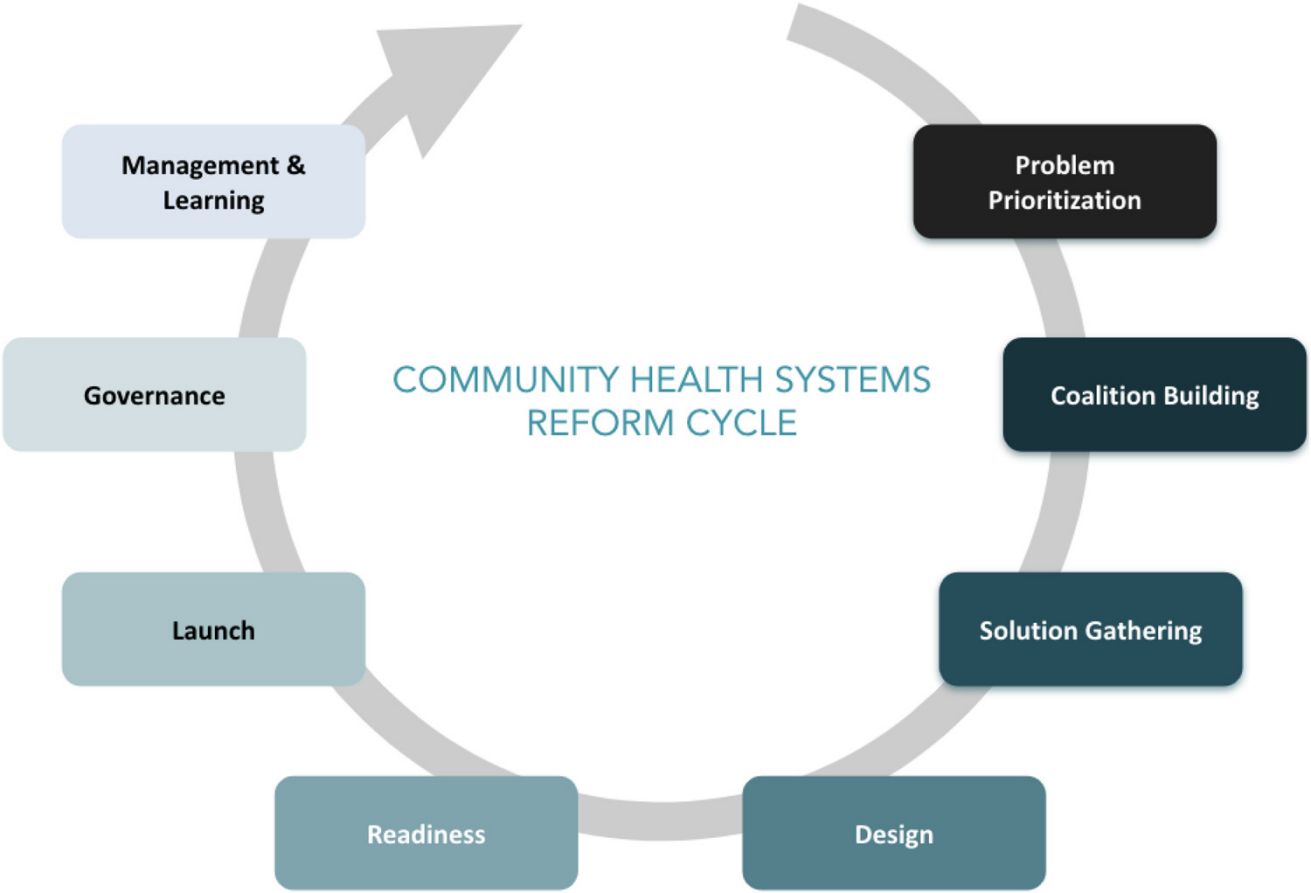
The Community Health Systems Reform Cycle

**Haiti Prioritized Community Health Institutionalization to Expand Primary Health Care.** Haiti's history illustrates how an urgent health sector problem—in this case, extending primary care to all citizens—was used to motivate prioritizing community health institutionalization. As Haiti was rebuilding the health system after the devastation of the 2010 earthquake, the government emphasized that weak health system infrastructure, an insufficient health workforce, and lack of primary health care of good quality were the core challenges driving some of the worst health outcomes in the Western Hemisphere. The Ministry of Public Health and Population (MSPP) and other stakeholders recognized the success of CHW programs in Haiti —ranging from HIV accompagnateurs, to women's health agents—at providing quality essential care services. However, the success of these programs was limited due to their fragmentation. While the government had defined an official cadre of community health agents (agents de santé communautaire [ASC]), in practice, they were primarily recruited and supported through NGO-run and verticalized programs that lacked standardization and only served a fraction of the Haitian population. Therefore, MSPP and its partners prioritized the need to create a unified, national cadre that could extend essential care services across Haiti and be sustained by the MSPP. Key to the government's vision was a transition from overlapping, disease-specific ASCs to multipurpose ASCs offering a standardized package of services. The government termed these multipurpose ASCs as polyvalent ASC (ASCP), drawing from collaboration with the governments of Cuba and Brazil as part of their commitments toward the earthquake response.

The MSPP recognized that a critical first step toward reform would be to better understand the challenges with Haiti's previous community health efforts and align stakeholders behind its vision. Between 2015 and 2019, the MSPP mobilized resources from funders and partners, such as USAID, UNICEF, the Global Fund, the World Bank, and Zanmi Lasante, to organize a series of activities to identify pain points and areas for reform. For example, the MSPP sought to develop a stronger understanding of the status of CHWs in Haiti. Leaders were concerned that there were significant overlaps and gaps in coverage that contributed to inefficient use of scarce resources. To gather the data necessary to address the problem, the MSPP and its partners mapped the distribution of CHWs (ASCs and ASCPs) across the country and calculated the ratios of CHWs to population by geographic area. This information is now helping the MSPP to realign the community health workforce, inform the national budgeting process, review the national community health strategic plan, and revise the curriculum for ASCP.

To demonstrate early national commitment to community health services and ensure a cohesive coalition could be assembled, the MSPP's leadership took early steps such as integrating a percentage of the ASCPs into the national budget. Reinvigorating that problem prioritization process was essential to help inform subsequent stages of reform, in particular reconvening a coalition and the design choices that would need to be considered within the policy, strategy, and program package.

The careful construction and maintenance of a winning coalition connects the priority problems with actors who can influence the health system throughout all stages of reform.

#### Coalition Building

In the coalition building stage, key stakeholders, organizations, and individuals (“coalitions”) are brought together to collectively effect change. A compelling problem at the right moment galvanizes a winning coalition, while a tepid problem quickly loses momentum. The careful construction and maintenance of a winning coalition connects the priority problems with actors who can influence the health system throughout all stages of reform. Successful coalitions are typically anchored around a high-level champion (often a minister-level official), and particular attention should be given to bringing in and consistently syncing with “well-networked health champions and strong national advocacy institutions.”^20^^,^^21^ Political economy tools or influence mapping may help identify who is needed for the coalition, what roles they play, and opposing interests. Coalition builders have the goal of crafting an “authorizing environment” for decision making that encourages experimentation and “positive deviance” and engaging broad sets of agents to ensure that reforms are viable, legitimate, and relevant.^13^

**Mali Built a Coalition Through Local Actors, Savvy Recruitment, and Well-Functioning Coalition Structures**. As Mali worked to roll out its strategy for advancing essential community health services, community health leaders struggled to generate momentum for reform. In response, in June 2017, the National Federation of Community Health Associations, the Ministry of Health (MOH), and supporting partners launched the National Advocacy Coalition for essential community health services as a platform for directing attention to the need for community health service reform in Mali. The local reform actors found a compelling and shared problem—persistent gaps in the quality of community-based health services due to the lack of proper payment, education, and support of CHWs—and framed it to recruit other influential donor voices, including USAID and UNICEF. The actors also devoted resources to create and sustain the National Advocacy Coalition. Functioning coalition structures such as a steering committee and a technical committee continue to meet regularly. Since 2017, membership in the National Advocacy Coalition has grown every year, from 12 member organizations in 2016 to 24 by 2020. During that time, the National Advocacy Coalition emerged as a leading voice driving national efforts for the sustainability of the essential community health services strategy.

### Discovering What Is Possible: Solution Gathering, Design, and Readiness

Our second finding is that successfully scaled and institutionalized community health programs must be sourced from the existing capabilities, practices, partners, and resources within a health system through a process of collective discovery and negotiation.

Successfully scaled and institutionalized community health programs must be sourced from the existing capabilities, practices, partners, and resources within a health system through a process of collective discovery and negotiation.

It is no wonder that large-scale CHW programs struggle with quality when they look and perform nothing like they did at smaller scales of operation. Initially, small-scale programs are often designed and managed by external partners. Government engagement is inconsistent. However, subsequent larger-scale programs often emerge when partner-supported programs are transitioned to government. Key elements of effective CHW programs, such as supportive supervision or competency-based training,^5^ are missing from community health programs that have been scaled in recent years.^22^ In some cases, design features are removed from the program during policy development and scale-up—this occurred in Liberia when a peer supervisor cadre integral to the successful pilots was removed from the nationally scaled design—while in other cases, health systems fail to effectively execute program activities beyond their original context and size.^9^

Institutional reform literature explains why “pilots never fail, pilots never scale.”^23^ CHW program design often begins with external “experts” improving programs through a mostly technical lens but shielded from the social, political, institutional, and policy realities required at larger scale. Moreover, effective tactics to engage these broader constraints are near impossible to predict or design in advance, especially from the outside. The success of a new institution depends on factors as wide ranging as how central, local, and external bureaucracies interact, prevailing and dissenting cultural norms about change, and explicit or hidden agendas and power.^24^ These factors and their effect on how a given externally sourced institutional model will operate once scaled are often invisible to actors within the system, including actors directly implicated in or affected by that context. As a result, Andrews et al. noted that the process of arriving at [new institutions] matters more than the form for sustained functional success.^13^ Effective institutional reform efforts require that a committed coalition of stakeholders undertake a process of discovery and learning to identify constraints and capacities within the health system and test potential solutions to the prioritized problem, drawing on existing institutions and capabilities.

The next 3 stages of the reform cycle, solution gathering, design, and readiness, reflect this process of discovery. These stages turn the energy of a coalition into early action toward solutions. Doing so requires actors to build from a shared understanding of the problem to develop a shared vision of the future, making difficult trade-off decisions about how to get there acknowledging the starting place, and taking the first steps.

#### Solution Gathering

During the solution-gathering stage, reform coalitions develop a shared set of criteria or principles defining what is needed to address the prioritized problem. Then, armed with these principles, the reform coalition must cast a wide net to identify potential solutions, drawing proposed solutions from both within the reform coalition and outside of it. Good solutions tend to be (1) technically correct, (2) politically supported, and (3) administratively feasible. These and other criteria can be tested by gathering rapid feedback, via consultation, workshop, survey, or small experiments with a wide array of health sector stakeholders. A strong practice of soliciting feedback in this manner during the solution-gathering stage helps to both inform better design and legitimize the reform via early wins.

A strong practice of soliciting feedback during the solution-gathering stage helps to both inform better design and legitimize the reform via early wins.

**Kenya Reformers Collaborated on Gathering Solutions to Improve Community Health Strategy**. Kenya provides a strong example of how collaborative solution gathering by reformers can help to accelerate progress from problem prioritization to later stages of design and readiness. In 2016, Kenya embarked on a comprehensive reform of its community health system. These reforms were interlinked with both Kenya's devolution of governance and its broader primary health care and universal health care agendas. The MOH-led Community Health Steering Committee served as the focal point for guiding stakeholders from problem prioritization into the solution-gathering stage. The committee first framed their core reform problem; Kenya's lack of a community health policy was an institutional weakness that left counties without clear guidelines for funding and implementation decisions. In response, the committee revitalized the process for developing the community health policy and revising Kenya's community health strategy. The group defined clear principles for an ultimate solution; to be successful, any new guidelines would need to reflect the current status of community health in the country, link to the President's universal health coverage agenda, gain support from county governments, and build on available evidence and innovation.

In 2018, the community health steering committee launched a community health services evaluation with funding from UNICEF and guidance from technical committees. The evaluation used a systems approach to examine the status of community health services within the devolved context and evaluate selected health outcomes. It also documented case studies and best practices from different county models.

A notable strength identified by the evaluation—and one that has positioned Kenya's community health system to function effectively within an increasingly devolved context—is the prominent role of community policy and governance structures, such as community health committees. For example, in 2017, UNICEF Kenya supported Turkana County to introduce a redesigned and locally adapted community health structure, which placed a community health volunteer in every village. The volunteer moves with households in nomadic villages, connects with the health facility management committees, and establishes a sublocation corresponding with each community unit, ensuring access to political and administrative services.^25^

The evaluation also revealed strengths in certain counties' prioritization, investment, and planning for community health. Under devolution, county governments are free to set their own budget allocations for health. By comparing counties with high- and low-performing counties, reformers developed a stronger understanding of how prioritization of community health services was driving coverage and health outcomes. The assessment of Siaya County is particularly illustrative. Siaya's government made the country's highest level of financial investment in community health and translated this financing into institutional functions such as community health commodity security and the provision of regular monthly incentives for community health volunteers. As a result, Siaya county drastically outperformed low-investment counties as well as the national average.^25^

The findings from the evaluation positioned the steering committee to make clear cases for how community health reform could accelerate the country's health agenda and base policy choices in the redesign of a revitalized community health strategy on domestically proven best practices and solutions.

**Democratic Republic of the Congo Advanced Community Health Reform.** In recent years, the Democratic Republic of the Congo (DRC) has taken unprecedented steps to advance community health reform, marking a shift from earlier stages of problem prioritization and coalition building to solution gathering.

These emerging reforms stem, in large part, from lessons learned during the Millennium Development Goal (MDG) era. In 2013, leaders recognized that the country was not on track to meet health-related MDGs and took stock of approaches that had proven successful, such as Integrated Community Case Management of Childhood Illnesses (iCCM). They determined that community health would need to be a driver of any corrective action and placed community health at the center of a new MDG flagship program.^26^ While DRC ultimately fell short of targets, the program contributed to notable improvements in health outcomes and moved community health to the forefront of the health sector agenda. The program's evaluation decisively recommended that the government prioritize “institutional anchoring” of community health to scale and sustain results in the long term. In conjunction, the government issued a call to action that without significant reform, the country would risk not attaining the SDGs.^27^ This problem framing set the stage for two major milestones in 2016. For the first time, the government explicitly integrated community health into the National Health Development Plan 2016–2020 (recently reframed and extended into 2019–2022). As a complement, the government crafted a Community Participation Strategy, defining the community structures and cadres that form the foundation of DRC's community health system. Together, these documents created an unprecedented policy basis for further community health reform.

However, to secure the place of community health and improve the effectiveness of its implementation, reformers recognized the need to assess the country's community health landscape, gather learning across a constellation of programming, and align resources and operational capacity behind a shared set of priorities. This would be no small task. For years, myriad normative documents in parallel technical areas had enabled a fragmented community health implementation environment. Further, those experiences were not sufficiently monitored, evaluated, or disseminated, thereby complicating attempts to distill and integrate best practice.

Accordingly, the MOH sought to address a core obstacle: the lack of a national, unified Community Health Strategic Plan (CHSP). In late 2017, the MOH mobilized a coalition, chaired by a technical committee of key stakeholders, to lead a participatory process of problem prioritization, coalition building, and solution gathering. The coalition worked across the layers of the health system to conduct stakeholder mapping exercises, desk reviews, key informant interviews and focus groups, and validation workshops. The result is a comprehensive situational analysis; strategic, operational monitoring and evaluation framework; and preliminary budget that are grounded in existing practice but present ambitious reform. At its core, the CHSP presents a set of solutions aimed at establishing a more harmonized, efficient, and effective community health system that is grounded in community engagement and aligns resources and actors. The institutionalization of these reforms will depend largely on a successful transition into later stages of the reform cycle.

#### Design

In the design stage, the reform coalition connects the policy or program reform goals that have been drawn from the prioritized problem (e.g., increased service coverage) with intervention designs (e.g., CHW recruitment and training). These designs, sourced via the solution-gathering process, may include new innovations, expansions of existing innovations, or revisions to programs already at some level of scale and institutionalization. Critically, stakeholders should ask themselves how the proposed interventions will function within the current system. At this stage, reformers must find a balance between pushing the system to develop new capabilities that address the prioritized problem, and exercising caution to avoid “premature load bearing,” where new program designs are overly optimistic about the existing technical, political, and operational capabilities within the health system and therefore fail to deliver the expected results. The ExpandNet framework provides a useful set of key areas of capacity inquiry: technical skills, training, logistics and supplies, supervision, leadership and coordination, monitoring and evaluation, physical facilities and equipment, values supportive of the innovation, human resources, and a necessary policy framework.^28^ This is also the stage of the process where coalition actors clarify the answer to key planning questions: What will be required of government, of partners, or of other technical institutions to implement the new design? Is there a need for one or more intermediary organizations to support the scaling up process alongside the government? What organizational or structural changes will be required to implement and roll out the model?

In the design stage, reformers must find a balance between pushing the system to develop new capabilities that address the problem and exercising caution to avoid being overly optimistic about existing capabilities.

Often in LMICs, community health impact is conceptualized as the result of community health “projects.” However, designers would be encouraged to think early on how the “project” evolves into an institutionalized, routine part of the health system^24^^,^^29^:

*Delivery at scale is not a gigantic project or a series of projects. We need to plan for millions, not thousands; for uncontrolled, not controlled, settings; for generations, not for 5 years; and for addressing, not working around, political and market realities.*

**Liberia Designed a New Community Health Program.** The structured, multistakeholder process used in Liberia in 2015 to design a new community health program illustrates the impact of a design process that builds from a well-constructed problem, considers the capacities of the existing system, and involves key health system stakeholders. As the Ebola outbreak was coming increasingly under control, Liberia set out to build the country's first national, incentivized CHW program. Effectively building the program from scratch required a comprehensive and data driven process to identify policy and design considerations for an initiative intended to address the dramatic health service gap faced by the country's most remote communities.

Picking up on the political momentum generated by the President's calls for reform, the Minister of Health revitalized a core steering committee called the Community Health Technical Working Group, composed of government, technical, and donor stakeholders, ensuring perspectives were diverse and contributed to an aligned vision, and that actors with a critical stake in the functioning of the health system were given a forum for collaboration. The working group was responsible for setting the vision of the planned CHW program, providing leadership on the institutional, system, and operational decisions that went into the design of the program.

Liberia reached a major milestone in 2016, when the working group finalized the National Community Health Policy and the Minister of Health approved it, establishing a national community health assistant (CHA) program. This marked a critical transition from policy to program design; the reform team shifted its attention to the development of a CHA training curriculum, supervision and information systems, recruitment and human resources standards, supply chain processes and a comprehensive costing of the program to inform resource mobilization. The working group established a set of subgroups to help drive this detailed design process, recognizing that the integration and harmonization of historically fragmented systems would need to be considered. These subgroups included training and supervision, community based information systems, supply chain, and human resources for health.

Although each subgroup took its own form and function, they shared some key components to the design process: the review and deliberation of key design considerations, informed by pilot projects, evidence and learning from implementation experiences, and best practices across key stakeholders; the assessment of the operational and scale feasibility of each element of the program; and the development of a management and sustainability strategy, including resource mapping and costing to inform how program rollout would occur.

### Readiness

During the program readiness stage, health systems actors align the necessary resources for launch. These include financial, material, human resources, programmatic, planning, and political commitments in service of reforming the system (often launching or expanding a program). In effect, the reform coalition must ensure there is a clear “launch” plan that applies strong planning and management tools to coordinate rollout of the new institution, including action plans, budgets, defined responsibilities among actors, coordination mechanisms for the coalition, governance structures, a monitoring plan, and a troubleshooting process.^30^ During the readiness stage, actors should begin anticipating the eventual transition of partner-delivered programming to the government or other permanent institutions once the institution is operating at scale. This might involve aiming to frame the financial resources of the new program within a budget envelope that the government can realistically finance, even if it means not having the “perfect” program.^31^ This is also a critical moment in the process for advocacy events that illustrate sustained political support, such as policy dissemination or program launch events.

**Uganda “Built” Readiness to Scale Community Health Nationally.** Uganda's recent progress in scaling community health is illustrative of this critical stage. In 2001, Uganda established village health teams (VHTs) to bridge the health service delivery gap into communities and households. Since then, an estimated 180,000 VHTs have been deployed across the country. However, after a 2014/2015 assessment found a number of critical challenges with the VHT program, a policy reform process led ultimately to new community health extension worker (CHEW) policy.

During the readiness stage, actors should begin anticipating the eventual transition of partner-delivered programming to the government or other permanent institutions once the institution is operating at scale.

In 2018, in anticipation of this new policy, the Ugandan MOH conducted district readiness assessments in 13 districts. The assessment identified key intervention changes and assessed areas such as current knowledge of health workforce, availability of health workforce, familiarity with e-health technologies, existing supervision practices, and existence of health unit management committees, among other things. The assessment revealed key challenges prior to implementation that were otherwise difficult to anticipate in design. For example, certain districts lacked a biostatistician which would make data reporting difficult and other districts had broken referral systems. This district readiness assessment and other activities illustrate critical steps of the program readiness stage: socializing changes to the health system, communicating role transitions, and identifying potential challenges to the change early in the process of rollout. Although readiness assessments or evaluations of programs are common in the research world, rarely are they explicitly connected to desired policy changes.

In 2019, despite encountering political setbacks in securing approval for the CHEW policy, the Uganda MOH continued to build “readiness” for implementing reforms of community health institutions. Even in the absence of a new official policy, the reform-minded stakeholders (including the MOH, donors, and implementing partners) identified areas ripe for reform and critical for policy change and documented the main priorities for the Ugandan community health system in a Community Health Roadmap.* These priorities include resource mobilization and costing the community health strategy, leadership and governance, multisectoral collaboration, supervision and motivation, investment in technology such as digital health, supply chain, and community engagement. In 2019/20, the Uganda MOH, in collaboration with partners, has advanced several of these priorities such as integration of the community health supply chain system and inclusion of community level data into national health information systems, such as DHIS2. Uganda's experience with the Community Health Roadmap demonstrates a key element of program readiness–taking a systems integration lens to identify what capabilities need to be marshalled or strengthened to pave the way for upcoming program or policy changes.

### Consolidating Progress and Laying the Foundation for Future Work: Launch, Governance, and Management and Learning

Our final finding from observation of efforts to scale and integrate community health programs is that successfully building these programs is an iterative, cyclical process. Reform efforts should proceed in a manner that anticipates this ongoing nature, rather than expecting scale-up to be successfully “completed.”

Reform efforts should proceed in a manner that anticipates an iterative, cyclical process.

This understanding is embedded in the final 3 stages of the reform process: launch, governance, and management and learning. During these last stages, the system and its actors are building processes to progressively extend implementation of the new institution to a greater portion of the health system, continuously increase capability of the actors implementing the institution, improve quality of the services delivered, and adapt the institutional design to new realities and lessons learned.

As reflected in our characterization of a reform cycle, we note that none of these stages are implemented in a necessarily linear or sequential order. This is especially true with the following 3 stages, during which launch, governance, and management and learning are likely all happening at the same time.

#### Launch

During the program launch stage, reforms are launched and actors take on new roles and responsibilities. Effective approaches reflect the understanding that launching a reform is not simply “implementing a new plan,” but that actors in the system are transitioning from one reality or identity to a new one. Reformers must recognize that actors in the system lose or let go of previous identities embedded in prior practices. For example, a new CHW might have previously felt confident as a high-performing community health volunteer, or a vertical program might lose control as it is rolled into a new CHW program platform. Launch requires intentional management of this transition via orienting stakeholders amidst the uncertainty of change, sourcing frequent feedback, celebrating early wins, and reminding stakeholders of the ultimate goal. Building on the socialization aspects of the program readiness stage, actors across the system are trained, equipped, and asked to begin adopting their new roles. Challenges in implementation should be expected, and troubleshooting systems should be set up to address emergent gaps or problems. Supervision, performance management, and monitoring systems are supported to reinforce quality and provide critical information about the performance of the reforms within the system.

Effective approaches reflect the understanding that launching a reform is not simply “implementing a new plan,” but that actors in the system are transitioning from one reality or identity to a new one.

**Mali Launched a CHW Remuneration Program**. Mali's journey toward paying CHW salaries illustrates a path from problem framing through program launch driven by local actors with appropriate support. A group of community health advocates coalesced around addressing the financing gap for the essential community health services strategy and the lack of sustainable payment mechanisms for CHW salaries. The MOH first conducted a costing analysis in 2016 and 2017 through the USAID-funded Health Policy Plus (HP+) that highlighted substantial challenges to understanding the costs of the essential community health services strategy, due to fragmentation, inaccurate information, and lack of centralized information.^32^ This key learning led to further research that found the overall cost of community health programming to be approximately US$13.7 million per year and that the majority of CHWs were operating informally and with inconsistent payment. This evidence helped frame the National Advocacy Coalition's push to increase the share of government contribution.

As the CHW payment issue was gaining traction, in September to October 2016, the Ministry of Health and Public Hygiene assembled a multisectoral group of experts to understand the legal constraints to paying CHWs as civil servants. Both the HP+ situation analysis and further inquiry into legal status were steps in solution gathering and program readiness–attempts to probe where capabilities in the health system lie and uncover the changes necessary to move reform forward.

In 2018, community health reformers further created and sustained the window of opportunity. Community health stakeholders had repeatedly held sessions with the parliamentarians on the importance of the essential community health services strategy as a whole and resource mobilization for CHW salaries specifically. In April 2018, the National Advocacy Coalition and the National Assembly's Health Commission organized testimonies from a CHW, a mother, a village chief, the president of a civil society organization, and a district health director to highlight the challenge to essential community health services sustainability if CHW salaries were not paid with domestic resources. In response, the National Assembly recommended initiating a bill to integrate CHWs as civil servants, an advocacy win that was broadcast on national television.

To turn this new priority into reality, the National Advocacy Coalition in Mali worked with the government, donors, and implementing partners to gather solutions, design, build readiness, and launch this new policy. The heart of the National Advocacy Coalition's goals was to persuade the government to provide a specific budget line to municipalities for the payment of CHW salaries. The National Advocacy Coalition convened all parties to identify mechanisms for paying CHWs through commune budgets and drafted a service contract between CHWs and community-level local authorities. The group identified Mali's Kadiolo district, where Save the Children had already been working with local actors since 2014 to shift CHW salaries to local budgets, as an opportunity to build off existing experience in new reform.

As part of program readiness planning, the National Advocacy Coalition identified the needed changes in local roles and ensured that parties were equipped to step into the new roles. This included training for local authorities on budget analysis, monitoring health expenditures, and holding roundtables to mobilize funds. Moreover, the mayors agreed to a gradual transition of financial ownership over 3 years (from 50% to 100%), which also allowed for actors to gradually build experience and responsibility for the reformed institution. Lastly, the Minister of Health visited Kadiolo just after the mayors committed to taking full responsibility for CHW salaries, which showed the Malian government's commitment to this reform and sustained momentum for these changes.

#### Governance

The governance of the system, as used here, refers to the set of rules (formal and informal) and relationships among actors that allow for collective action and decision making, including setting of strategic direction, creating an enabling environment, and overseeing execution.^33^^,^^34^ During this stage, actors establish systems and methods by which a program's strategy and plan will be defined, authorized, and monitored.^35^ The establishment of formal governance often accompanies the transition from outside reform priority to institutional adoption. As financial commitment to funding CHWs may not be institutionalized across a particular health system, it is important to plan for the governance systems for payment and human resources management to reduce silos and fragmentation during the implementation phase.

It's important to plan for the governance systems for payment and human resources management to reduce silos and fragmentation during implementation.

**Bangladesh Improved Governance to Support Community-Based Primary Health Care Services.** With a rich history of CHWs as a key pathway to improve primary health and family planning priorities in the 1970s and 1980s, and a prolonged national rollout of a Community Based Health Care program over the last few decades, Bangladesh has recently turned its focus into improving program management and governance to support the ongoing implementation and quality of its community based primary health care services. However, as country of 161 million people, Bangladesh still faces a particularly complex challenge in constructing its health governance structures. Moreover, by some estimates, the public sector provides less than 20% of curative services, and the rest are provided by complementary private or NGO service providers.^36^ To manage this, the government has instituted multiple layers of governance systems to encourage formal and informal actors to swim in the same direction. Bangladesh's experience illustrates the critical governance functions of defining roles and setting goals for the sector and its associated challenges.

At a national level, Bangladesh has used a sector-wide approach (SWAp) for sector planning since 1998 and continues to do so in each subsequent health sector strategy cycle. This approach emphasizes holistic government defined operational plans that donor and NGO partners help execute. For example, the 1998 SWAp replaced 128 discrete projects under the Ministry of Health and Family Welfare. The SWAps “facilitated the alignment of funding and technical support around national priorities, and improved the government's role in program design as well as in implementation and development partner coordination.”^37^ This governance approach, like any, is not without its challenges; in recent years, the distinct operational plans of different line ministries have generated sector fragmentation, especially given that governance of community health sector activities is held in 2 different directorates (family planning and general health services).

At a subnational level, upazila (subdistrict) health councils and ward-level community clinics provide governance support. The fourth national health plan also reinforced commitment to the vision of the community clinic as the basic unit of the primary health care system and extended the scope of the community program to include the provision of essential health services from all upazila health facilities.^36^ With this, the program aimed to strengthen health system integration across community, upazila, and district levels. When properly implemented, the community clinics and accompanying community groups serve as effective forums for coordination, where leaders or community groups from within a clinic's catchment can exchange information with other levels of the health systems and define their own needs. However, many community clinics still struggle with the coordination and planning functions of governance.

#### Management and Learning

During the management and learning stage, actors implement reformed policies and programs and utilize learning and data to inform improved performance of the system. Key stakeholders identify gaps in implementation and enforce adherence to established standards. During this time, gaps or obstacles are addressed to achieve strong performance.

**Bangladesh Evolved Community Health Service Implementation Using Program Learning and Data.** Bangladesh's history with community health services also illustrates the management and learning elements needed for progressively advancing reform. Over 4 decades, Bangladesh's community health services have evolved from including family planning services in the 1970s to adding oral rehydration and immunization services in the 1980s to additional broadening of the service package (maternal health, other essential services) and greater integration into the primary health care system with community clinics over the past 30 years. Each of these changes was responsive to data on health challenges. Much of this also benefited from the SWAp governance framework's integration of inputs across program monitoring data, research institutions like the International Centre for Diarrheal Disease Research, various government agencies, and implementing partners like BRAC. These partnerships allowed Bangladesh to bring together sources of programmatic data and implementation research findings, supporting effective program management and learning, ongoing identification of gaps in implementation, awareness of changing context, wide scope of evidence to inform change, and open channels for innovation diffusion.

**Liberia Established Quarterly Collaborative Forum to Review Data and Drive Decision Making.** The Liberia MOH also integrated a set of comprehensive adaptive management and learning practices across the country, immediately following the launch of the national CHA program, during which the MOH and partners trained and deployed over 3,000 CHAs across 14 of 15 of Liberia's counties. As each of the 15 counties in Liberia receives varying levels of financial and technical support from a range of donors and NGO partners, coordination has become increasingly challenging and even more critical in order to maintain government ownership and the quality of implementation across counties. To sustain program management and learning in this complex landscape, the MOH established quarterly review meetings to bring key stakeholders together in a collaborative forum to review existing and new data to drive discussion and adaptive decision making.

During these review meetings, the MOH, national and subnational government stakeholders, donors, and partners review program performance and develop policy and implementation adaptations that are informed by the program's successes and challenges in real time.

Quarterly meetings have become an essential part of the institutional structure of the national CHA program. This convening promotes government ownership and allows the MOH to cultivate a culture of continuous learning and adaptive management where all stakeholders are aligned and accountable to set performance indicators, work together to identify persistent problems, and commit to adopting successful implementation practices that address them. The result is significantly improved program management and learning, where the MOH is able to lead other stakeholders in analyzing data to prioritize unresolved problems, plan experiments to test potential solutions, and develop action plans for actors at all levels to ensure solutions are scaled. This illustrates how effective management and learning practices allow for continuous cycles of reform, with each newly scaled institutional component (e.g., standardized CHW cadre) revealing new challenges and opportunities for further reform (e.g., improving accuracy and timeliness of reporting via digitization of community-based health information systems).

## CONCLUSION

Taken together, these 8 stages offer a roadmap for governments, health sector partners, and others seeking to support the scale-up and institutionalization of CHW programs. The cycle can be used diagnostically—as a framework for assessing whether would-be reformers have addressed the key considerations critical to success—or as a planning tool for focusing the efforts of health sector stakeholders seeking to make change. For community health programs that are already underway, the reform cycle considerations can illustrate where further efforts should focus. Additionally, reformers entering a new stage of a reform process can use the key considerations associated with the stages of the reform cycle to prioritize their work. These broadly align with lessons from exemplar community health countries in taking a problem-driven approach, cultivating political will, and building government-led coalitions.^38^

These 8 stages offer a roadmap for those seeking to support the scale-up and institutionalization of CHW programs.

This work should be situated in the context of other complementary trends and current initiatives. Partners to the SDG Global Action Plan's PHC Accelerator may use this framework and supplemental tactics to move the levers of the PHC system. As countries and partners prepare for the upcoming Institutionalizing Community Health Conference 2.0, this can inform diagnostics and targeted planning for reform efforts. In many countries, the COVID-19 pandemic has both exacerbated existing inequities and fragilities in the health system, while accelerating windows for opportunity for reform — the Reform Cycle can help make the most of these opportunities.^39^

This framework is based on the premise that building and sustaining community health programs requires employing the tools of institutional reform. This premise should be examined through further research that aims to do the following:

- Revisit failed or partially successful community health efforts and assess whether gaps in reform process as outlined in this cycle contributed to the suboptimal outcome.
- Test applications of the reform cycle in new and ongoing reform efforts by encouraging coalitions of governments, development partners, and donors to experiment with the reform cycle as a diagnostic and planning tool.
- Spur greater investment by governments, development partners, and donors in the requisite governance, programmatic monitoring, and implementation research efforts to provide timely feedback on reform processes. Given the potential contribution of community health to achieving global and country-level goals for extending PHC and achieving universal health coverage, a deeper investment in understanding reform processes should be reflected in health sector budgets and investment plans, as part of advancing those goals.
- Compare the reform cycle with systems change and reform frameworks from other sectors (e.g., collective impact models).

We hope that application of this framework over time will result in more effective integration and institutionalization of community health programs that support CHWs to provide essential health services to the most under-served populations globally.

## Supplementary Material

20-00429-Chen-Supplement.pdf

## Footnotes

* All countries profiled, except Bangladesh, developed a Community Health Roadmap, see https://www.communityhealthroadmap.org/.

## Notes

**Peer Reviewed**
