# Supplementary material for: The Community Health Systems Reform Cycle: Strengthening the Integration of Community Health Worker Programs Through an Institutional Reform Perspective
Source: Glob Health Sci Pract. 2021 Mar 15;9(Suppl 1):S32–46. doi: 10.9745/GHSP-D-20-00429 (PMC7971380; doi:10.9745/GHSP-D-20-00429)
Supplement: 20-00429-Chen-Supplement.pdf [file 20-00429-Chen-Supplement.pdf]

Supplement to: Chen N, Raghavan M, Albert J. The community health systems reform cycle: strengthening the integration of community health worker programs through an institutional reform perspective. *Glob Health Sci Pract*. 2021;9(Suppl 1). https://doi.org/10.9745/GHSP-D-20-00429

The Process of Developing a Conceptual Framework for Community Health Systems Reform

The framework in this article was workshopped over 3 group technical advisory sessions including practitioners, researchers, donors, and representatives of community voices, and numerous 1-on-1 discussions. The framework was further workshopped during country research trips that produced the vignettes in this article. These examples were generated through extensive desk review and engagement with country stakeholders. Insights and country stakeholder recommendations from this process served not only to validate the reform cycle framework, but also to highlight ways in which the framework can help trace powerful narratives of reform.

In addition to the above, this article draws heavily from literature on policy reform, change management, institutional development, systems thinking, and advocacy theories, to identify principles from these disciplines that might be applied to scaling and integrating community health systems. These principles are applied more directly in the following sections, however, we describe some overarching themes.

- Institutional reform efforts, like community health reform, are complex, adaptive processes characterized by: (1) uncertainty on how exactly to achieve success in a given context, (2) divergence among key actors on the problem/solutions, and (3) knowledge and capacities that are distributed across these actors.[i],[ii] This complexity is an underlying challenge to "policy implementation and bureaucratic performance."[iii] In community health, the horizontal nature of community health interventions requires special attention to integrating across historically vertical actors (e.g., malaria, family planning, disease surveillance). Interventions should be more adaptive by design and rapidly responsive to new information.

- The systems thinking literature de-emphasizes any one actor's ability to directly engineer large-scale change, but rather focus on "creating the conditions that can produce change and that can eventually cause change to be self-sustaining,"[iv] and importantly on empowering local actors that will lead and sustain that change.[v] Community health reform is distinctive in that the workforce themselves are part of the distributed leadership of reform; they deliver services and promote change in the local systems. Leaders of community health reform efforts must rely on networks of actors beyond one's direct control.

- These efforts are nonlinear and investments payoff in unexpected ways; see for example a U.S. Agency for International Development anti-corruption project that "failed" during its project period, but where the program manager "continued to dedicate his life to good governance, founded an influential nonprofit, brought many of the anti-corruption ideas into government after the Rose Revolution, and is now Speaker of the Parliament."[vi]

Supplement to: Chen N, Raghavan M, Albert J. The community health systems reform cycle: strengthening the integration of community health worker programs through an institutional reform perspective. *Glob Health Sci Pract*. 2021;9(Suppl 1). https://doi.org/10.9745/GHSP-D-20-00429

Community Health Systems Reform Cycle: Milestones, Considerations and Tools

Building Political Will: Problem Prioritization & Coalition Building

Problem Prioritization

During the stage of problem prioritization, local reform actors diagnose and frame a compelling problem or opportunity that convinces critical stakeholders of the need for action. Compelling problems harness windows of opportunity, reflect locally defined challenges, and are framed to bring together individuals and organizations that can collectively affect change. A compelling problem at the right moment can galvanize a winning coalition - while a tepid problem can quickly lose momentum. The careful construction and maintenance of a winning coalition connects the priority problems with actors who can influence the health system to change throughout all stages of reform.

Cycle Stage	Problem Prioritization & Diagnosis
Milestones	During this stage, actors diagnose and frame a compelling problem or opportunity that sets the stage for the rest of the cycle. Critical milestones are:<br>• A meaningful and relevant problem has been identified and framed<br>• Pain points and unmet needs have been defined. Where possible, these are connected to priority areas for reform<br>• Relevant actors acknowledge the need for reform within the community health system, while committing towards a joint vision for addressing gaps**.**
Key Considerations from a Reform Perspective	**Windows of Opportunity**. Consider which windows of opportunity for change are open or closed. Often, windows are opened by political or economic shocks, routine changes like administration

	transitions, while in other cases actors can frame facts as urgent windows in which a problem can be prioritized.[1]<br><br>**Problems as Opportunities**. As stakeholders deconstruct problems, you'll likely find that most community health challenges (e.g. health access) are made up of many sub-causes (supply chain, political blockage, finance fragmentation). Each of these are also different opportunities for change, with potential stakeholder alignments behind them.<br><br>**Key Stakeholders**. Notice who are the key stakeholders defining the problem, and how they may shape the problem's scope. Problem prioritization often focuses on health data like disease burden, but consider also political data such as how key stakeholders might frame the issue.<br><br>**Locally Defined and Problem Driven.** Where the problem/opportunity can be locally defined by influential actors, these will have early gains in coalition building and stand a greater chance for institutional adoption later. During early stages, focus on actually solving specific problems as the goal (rather than introducing a pre-designed solution).[2]<br><br>**Why no change?** Consider why – given that the gap in service provision or public sector capability is recognized as a problem by at least some stakeholders -- it has not already been addressed through institutional change. This may inform a reframing of a problem to increase likelihood of action. Political economy tools are helpful.
Resources	• Constructing and Deconstructing Problems exercises from Building State Capability book (Chapter 7)<br>• The CHW AIM Tool is a useful starting place for Community Health programs to identify current level of capability and likely constraints on further program effectiveness<br>• DFID's Drivers of Change approach

[1] Baumgartner FR, Jones BD. *Agendas and Instability in American Politics, Second Edition*.; 2013. doi:10.7208/chicago/9780226039534.001.0001; Perry J, Kingdon JW. Agendas, Alternatives, and Public Policies. *J Policy Anal Manag*. Published online 1985. doi:10.2307/3323801.
[2] Andrews M, Pritchett L, Woolcock M. Building State Capability: Evidence, Analysis, Action. *Oxford University Press*. 2017. doi:10.1093/acprof:oso/9780198747482.001.0001.

	• USAID's Applied Political Economy Analysis

Coalition Building

During this stage, relevant actors acknowledge the need for community health systems reform and begin creating the formal and informal bonds to work together. Coalition as defined here are "individuals, groups or organizations that come together to achieve social, political and economic goals that they would not be able to achieve on their own."[3],[4]

Cycle Stage	Coalition Building
Milestones	• Group is formed around a compelling problem or vision<br>• Members understand the group and individual roles and goals<br>• Size and composition of the group is fit for purpose<br>• Diverse membership that can fill critical roles for reform (e.g. leaders, connectors, gatekeepers, donors, enablers, change champions and links to key players outside the coalition).<br><br>The coalition may convened to address reforms including: (1) address an urgent situation; (2) to empower elements of the community - or the community as a whole - to take control of its future; (3) to actually obtain or provide services; (4) to bring about more effective and efficient delivery of programs and eliminate any unnecessary duplication of effort; (5) To pool resources; (6) To increase communication among groups and break down stereotypes; (7) To plan and launch community-wide

[3] DLP. Coalitions in the Politics of Development. Findings, insights and guidance from the DLP Coalitions Workshop, Sydney, 15-16 February http://www.mspguide.org/sites/default/files/resource/dlp_coalitions_in_the_politics_of_development.pdf

[4] University of Kansas, Center for Community Health and Development- Community Toolbox: "Starting a Coalition" 2020. https://ctb.ku.edu/en/table-of-contents/assessment/promotion-strategies/start-a-coaltion/main

	initiatives on a variety of issues; (8) To develop and use political clout to gain services or other benefits for the community; (9) To create long-term, permanent social change.[5]
Key Considerations from a Reform Perspective	**Change Champion.** Particularly attention should be given to bringing in and consistently syncing with high-level champions (often a minister). "Well networked health champions and strong national advocacy institutions" embedded in local contexts are key to executing and sustaining reform.[6]<br><br>**Political Economy Analysis.** Political economy tools or influence mapping may help identify who is needed for the coalition, what roles they play, and opposing interests. Mapping out who may lose from this change is often forgotten but a critical and necessary step. This informs how to creation of an *"authorizing environment"* for decision-making that encourages experimentation and *"positive deviance"*; and...engage broad sets of agents to ensure that reforms are viable, legitimate, and relevant. [7]<br><br>**Consider interests of the coalition not just the "program"**. In many cases, the aligned interest of a coalition are not just a community health "program", but what that program promises to deliver (e.g., health results, better coordination). Consider how those larger objectives can create a wider coalition, and more political clout.
Resources	• University of Kansas, Community Toolbox*: Building a Coalition*<br>• Coalition Effectiveness Checklist<br>• Building an Advocacy Coalition<br>• Working Upstream: Skills for Social Change<br>• USAID - Applied Political Economy Analysis toolkit

[5] University of Kansas, Center for Community Health and Development- Community Toolbox: "Starting a Coalition" 2020. https://ctb.ku.edu/en/table-of-contents/assessment/promotion-strategies/start-a-coaltion/main
[6] Murunga V, Musila N, Oronje R, Zulu E. The role of political will and commitment in improving access to family planning in Africa. In: *Policy, Reproduction, and Sexual Health*. 2013; Shiftman J. Generating political priority for maternal mortality reduction in 5 developing countries. *Am J Public Health*. Published online 2007. doi:10.2105/AJPH.2006.095455
[7] Andrews M, Pritchett L, Woolcock M. Building State Capability: Evidence, Analysis, Action. *Oxford University Press*. 2017. doi:10.1093/acprof:oso/9780198747482.001.0001.

Supplement to: Chen N, Raghavan M, Albert J. The community health systems reform cycle: strengthening the integration of community health worker programs through an institutional reform perspective. *Glob Health Sci Pract*. 2021;9(Suppl 1). https://doi.org/10.9745/GHSP-D-20-00429

Discovering what is possible: Solution Gathering, Design, and Readiness

Solution Gathering

During the solution gathering stage, reformers develop a shared set of criteria or priorities based on the prioritized problem. Then, armed with these principles, the reform coalition must cast a wide net to identify potential solutions while maintaining an understanding of existing capabilities, and where possible, source rapid feedback on available ideas from a wide array of health sector stakeholders.

Cycle Stage	Solution Gathering
Milestones	• Criteria or priorities are developed to determine how to assess solutions<br>• Potential solutions are gathered, drawing from existing, local, and international ideas<br>• Where possible, specific ideas for reform are tested/piloted for effectiveness<br>• Promising solutions are prioritized for integration into the health system.
Key Considerations from Reform Perspective	**Criteria.** During this phase, it is important to understand where existing institutional capabilities and existing political will and buy-in can help narrow the focus of the solutions. Within the system, there are likely to be a number of proposed solutions- within the coalition of actors, or outside of it that would be considered. Good solutions tend to be (1) technically correct, (2) politically supported, and (3) administratively feasible.<br><br>**Rapid Feedback**. During early stages of solution gathering and design, reformers will often be designing in the abstract. If possible, gathering rapid feedback via consultation, workshop, survey, or small experiments help to both inform better design and legitimize the reform via early wins.<br><br>Features of scalable interventions may include:

Supplement to: Chen N, Raghavan M, Albert J. The community health systems reform cycle: strengthening the integration of community health worker programs through an institutional reform perspective. *Glob Health Sci Pract*. 2021;9(Suppl 1). https://doi.org/10.9745/GHSP-D-20-00429

	• Relevance & importance<br>• Effective & advantageous<br>• Observable benefits<br>• Acceptable to key stakeholders and community<br>• Simple & low cost<br>• Aligned with government priorities<br>• Harmonized with other peers and the coalition of actors<br>• Adaptable to different geographic and socio-economic contexts<br>• Low recurring costs or income generating
Resources	• Constructing and Deconstructing Problems exercises from f Building State Capability book (Chapter 7)<br>• Positive Deviance Field Guide

Design

In the Design stage, designers connect the policy or program goals that have been drawn from the prioritized problem (e.g., increased service coverage) with intervention designs (e.g., CHW recruitment and training). These designs, sourced via the solution gathering process, may include new innovations (often called pilots), expansions of existing innovations, or revisions to programs already at some level of scale and institutionalization. Critically, stakeholders should ask themselves how the proposed interventions will function within the current system. At this stage, reformers must find a balance between pushing the system to develop new capabilities that address the prioritized problem, and caution to avoid “premature load bearing,” where new program designs are overly optimistic about the existing technical, political, and operational capabilities within the health system and therefore fail to deliver the expected results.

Often in LMICs, community health impact is conceptualized as the result of community health “projects”, but designers would be encouraged to think early on how the “project” evolves into an institutionalized routine part of the health system - “Delivery at scale is

not a gigantic project or a series of projects. We need to plan for millions, not thousands; for uncontrolled, not controlled, settings; for generations, not for five years; and for addressing, not working around, political and market realities."[8]

Cycle Stage	Design
Milestones	• Key decisionmakers, inputters, and authorizers of the design/reform are identified. These may be a small group (like a village council for very local reform), or large multi-sectoral groups for national policy changes. All stakeholders are aware of their role in the design process<br>• From the solution gathering stage and other key informants, designers map and understand the different design choices to address the problem. Where possible evidence about the different design options and expected cost, impact, feasibility are identified<br>• Through consultations, workshops, and other forums, groups recommend design choices and decisionmakers are able to validate these choices.<br>• For the community health system, this stage is comprehensive can take long periods of time due to moving from a policy and strategy to the design of a community health program which can include training materials, costed operational plans, job descriptions, supervision tools and data collection systems, supply chain processes and planning documents to plan for launch of a program or initiative.
Key Considerations from a Reform Perspective	Design should take into consideration the functional capacities of the existing systems and assess the feasibility and tradeoffs between incremental improvement versus large leaps. Incremental success also provides quick-wins that build more buy-in and capabilities. Other factors may include staff and technical capacity, reputation, leadership, network, financial resources and management and governance oversight. During this time, key questions should also be discussed within the coalition leading the efforts on policy and program design:<br><br>• **Systems Functionality -** Consider does the community health system have system capacity needed for the design? The ExpandNet framework provides a useful set of key areas of capacity

[8] L. Cooley, J. Howard. Scale Up Sourcebook. *Innovations in Agriculture: Scaling Up to Reach Millions*. 2019. Available at http://docs.lib.purdue.edu/scaleup/sourcebook/book/1/ (2019); Woltering L, Fehlenberg K, Gerard B, Ubels J, Cooley L. Scaling – from "reaching many" to sustainable systems change at scale: A critical shift in mindset. *Agric Syst*. Published online 2019. doi:10.1016/j.agsy.2019.102652

	inquiry - technical skills, training, logistics/supplies, supervision, leadership/coordination, monitoring/evaluation, physical facilities and equipment, values supportive of the innovation, human resources, and necessary policy framework.[9] Similar questions are:<br>○ What are the institutional demands on the model- what will be required of government, of partners or other technical institutions?<br>○ Is there a need for one or more intermediary organizations to support the scaling up process alongside the government? What type of support is needed and who is best suited to support those roles?<br>○ What organizational or structural changes will be required- staffing, operations, to implement and rollout the model?<br>● **Experimentation and Iteration** - Traditional design invests a lot of resources upfront in the design, and only adapt to poor design years after. Consider how to build in more experimentation and iteration directly into the program. Consider running multiple designs at the same time, iterate design to allow solutions to emerge during implementation, build strong learning feedback systems.<br>● **Designing for the Future -** Have we designed with institutionalization, integration, and scale in mind? Is the intervention designed as a narrow-stand alone project?[10]<br>○ What changes are needed to make the model viable in other parts of the country?<br>○ Are there clear action steps in standing up, adopting, evolving, or transferring the model?<br>● **Political Will** - Does funding and political support exist to replicate the model on a large scale?<br>○ What design components were supported and by whom? What does this say about political support for scale?<br>○ What benefits of initial roll-out might change the support for this type of program?<br>○ What is the medium to long term sustainability strategy?
Resources	● Community Health Academy Systems Areas Tool and Health Systems Leadership Course<br>● Center for Global Impact- Policy Diagnostic Tool<br>● WHO ExpandNet

[9] WHO. Nine steps for developing a scaling-up strategy. *WHO*. 2010. Retrieved from http://www.who.int/reproductivehealth/publications/strategic_approach/9789241500319/en/

[10] Signs of this include - reliance on external financial and human resources, fixed time horizon, partnerships based on the project objectives and not the societal problem, shielding from “real world” constraints. Woltering, L., et al. (2019). Scaling – from “reaching many” to sustainable systems change at scale: A critical shift in mindset. Agricultural Systems, 176, 102652. https://doi.org/10.1016/j.agsy.2019.102652

Readiness

As the process enters the program readiness stage, health systems actors align the necessary resources for launch. These include financial, material, human resources, programmatic, planning, human, and political commitments in service of reforming the system (often launching or expanding a program).

Cycle Stage	Readiness
Milestones	• **Mobilize the financial Resources** and set up **financing mechanisms** as needed to ensure funds are available and can readily flow to the right actors for implementation.<br>• Translate program design is often translated into **costed operational plans and implementation guidance** documents at this stage.<br>• Ensure there is a clear **"launch" plan** in which there is strong planning and management tools to ensure rollout out happens in a coordinated and organized manner<br>• The coalition actors and champions **socialize, disseminate, and communicate** explicit support around the new program to actors who will need to play key roles in transition, roll-out, and maintenance. Ensure that actors across the spectrum are aware, bought-in, and have the right knowledge.<br>• Additionally, **investment plans for sustainable financing** are put in place and stakeholders are fully oriented on and resourced to fulfill new roles and responsibilities.<br>• Building on the design phase, this is also a good opportunity to **identify integration needs** across the health system or policy/protocol conflicts that will need to be addressed.
Key Considerations from a Reform Perspective	• **Be explicit about new responsibilities** - In addition to the launch plan, ensure other operational readiness exists - action plans in place, budgets in place, defined responsibilities amongst actors, coordination mechanisms setup, governance structures, monitoring plan, and trouble-shooting

	process.[11]<br>● **Buy-In and Advocacy -** Key advocacy activities should take place during this time- continuing to generate strong political will and signal to stakeholders that the coalition's support behind the program is consistent and strong<br>○ Dissemination and launch activities should be planned to sensitize and social stakeholders to the program launch, the benefits of the program and to create dialogue around the roles and responsibilities of an actor within the system<br>○ Identify stakeholders who might be resistant to the change, and develop ways to address that resistance<br>● **Contracting** - For designs that require contracting, consider ways to design the contract to be adaptive. E.g., focus more on outcomes, build in review and pivot periods, incorporate scenario planning.<br>● **Aid Effectiveness** - When mobilizing financial resources for a new reform, make choices that prioritize aid effectiveness<br>○ country ownership (take a country reform perspective, not project-based)<br>○ alignment (align to country targets and priorities)<br>○ harmonization (shared metrics, measurement frameworks, and coordination-governance)<br>○ transparency and accountability (shared monitoring frameworks and resource mapping)<br>○ Aid predictability<br>○ Civil society and engagement.[12]<br>● **Plan for Uptake and Transition -** Consider any anticipated transition of management or capabilities building early on.<br>○ As part of design and mobilization, aim to match the financial resources of the program with a budget envelope that government can take on - even if it means not having the "perfect" program.[13]<br>○ This is often a good stage to identify a joint learning agenda amongst actors. A learning agenda can also be a place to put areas of misalignment, uncertainty, or disagreement amongst the coalition that can be addressed later

[11] Milat AJ, Newson R, King L. *Increasing the Scale of Population Health Interventions: A Guide*.; 2014, available from: www.health.nsw.gov.au/research/Publications/scalability-guide.pdf
[12] Wickremasinghe D, Gautham M, Umar N, Berhanu D, Schellenberg J, Spicer N. " Its about the idea hitting the bull's eye": How aid effectiveness can catalyse the scale-up of health innovations. *Int J Heal Policy Manag*. Published online 2018. doi:10.15171/ijhpm.2018.08
[13] Spicer N, Hamza YA, Berhanu D, et al. "The development sector is a graveyard of pilot projects!" Six critical actions for externally funded implementers to foster scale-up of maternal and newborn health innovations in low and middle-income countries. *Global Health*. Published online 2018. doi:10.1186/s12992-018-0389-y

	• **Systems Integration -** Consider which parts of the reform will require changes in other parts of the system or immediate integration. This is a good opportunity to (1) identify low-hanging and easy integration opportunities, and (2) chart a roadmap for changes that may need to be designed in the future.
Resources	• MSH Community Health Costing Tool<br>• Financing Alliance for Health - Analytical Tools<br>• USAID CII's Ready, Set, Launch toolkit<br>• VillageReach- Transitioning Social Solutions to Government & Guidelines Request

Consolidating Progress and Laying the Foundation for Future work: Launch, Governance, and Management and Learning

Launch

During the program launch stage, reforms are launched and actors take on new roles and responsibilities. Effective launch practitioners understand that this is not simply "implementing a new plan," but that individuals in the plan are transitioning from one reality or identity to a new one. Launch benefits from intentional management of this change via - normalizing the uncertainty, frequent feedback, early wins, reminding of the goal. Building on the socialization aspects of the program readiness stage, actors across the system are trained, equipped, and begin adopting their new roles. Challenges in implementation are completely expected, and troubleshooting systems should be set up to address. Planned supervision, performance management, and monitoring systems are supported to reinforce quality and provide critical information about the performance of the reforms within the system.

Cycle Stage	Launch

Milestones	• **New Capabilities** - Actors from CHWs and supervisors to program managers and MOH staff acquire new skills and knowledge to execute their new roles.<br>• **Reorganization** - New processes, roles, and potentially organizational structures are identified, socialized, and then implemented. In some cases, this is a simple shift while others may take months.<br>• **Initial Implementation** - The program reform is implemented in target areas.<br>• **Rapid Results** - From initial implementation, results are collected to demonstrate momentum and identify challenges.<br>• **Rapid Feedback** - Particular attention paid to roll-out challenges to make shifts in design quickly.
Key Considerations from a Reform Perspective	• View program roll-out not as simply implementing the plan, but as a **transition from one reality to another** that benefits from active management - drawing from "change management" practices. Consider (1) how actors lose or let go of previous identities embedded in prior practices - e.g., a new CHW previously felt confident as a high-performing community health volunteer, vertical program losing control as it is rolled into a new platform CHW program, (2) how to maintain support for the new change during the messy transition period before full comfort with the new model - e.g., normalizing the uncertainty, frequent feedback, early wins, reminding of the goal.[14]<br>• **Frequent and consistent communication** with key actors during this time is key. In particular, engage with community actors who need to ultimately use, support, or allow the program[15] - conducting formative research earlier and continued socialization, engagement, and feedback throughout program design and execution. Pay attention to both traditional actors like leaders or existing community structures and those that may be historically excluded from participation (e.g., women, youth, other marginalized groups).<br>• As challenges are experienced, the coalition should **convene frequently to address any gaps arising during rollou**t, engaging with key stakeholders at national and sub national level to

[14] Bridges, W. 'Managing Transitions,' *Da Capo Lifelong Books*; 2009; WHO and USAID. A Guide for Fostering Change to Scale Up Effective Health Services. 2007.
[15] Spicer N, Hamza YA, Berhanu D, et al. "The development sector is a graveyard of pilot projects!" Six critical actions for externally funded implementers to foster scale-up of maternal and newborn health innovations in low and middle-income countries. *Global Health*. Published online 2018. doi:10.1186/s12992-018-0389-y

	provide support and guidance.<br>● Documentation and **learning plan**s should be put in place to ensure key successes, challenges are captured to assess fidelity and quality of program rollout
Resources	● USAID CII's Ready, Set, Launch toolkit<br>● Stages of Team Development Brief - Forming, Storming, Norming

Governance

The governance of the system, as used here, references the set of rules (formal and informal) and relationships among actors that allow for collective action and decision making - setting of strategic direction, creating an enabling environment, and overseeing execution.[16] During the program governance[17] stage, actors establish systems and methods by which a program's strategy and plan will be defined, authorized and monitored.

Cycle Stage	Governance
Milestones	● **Establish a Project Governance Framework** which includes key leadership and decision-making bodies and clear roles, responsibilities, and decision rights. Key actors within the system should be invited to participate or aware that a governance framework exists. Governance structures are established that can appropriately reflect and represent the system's interest in the program.

[16] Pyone T, Smith H, Van Den Broek N. Frameworks to assess health systems governance: A systematic review. *Health Policy Plan*. Published online 2017. doi:10.1093/heapol/czx007; USAID, March 2013. The eManager. Management Strategies for Improving Health Services. How to Govern the Health Sector and its Institutions Effectively. Available at: http://www.lmgforhealth.org/sites/default/files/eManager_How%20to%20Govern%20the%20Health%20 Sector_4.11.13_FINAL.pdf

[17] Grindle MS. Good enough governance: Poverty reduction and reform in developing countries. *Governance*. Published online 2004. doi:10.1111/j.0952-1895.2004.00256.x

	• **Define processes and systems** for risk and issue management; stakeholder engagement and cross functional communication. Building out the rules, expectations, meeting cadence, and supporting tools - e.g., mapping of actors and programs.<br>• **Execute the governance framework** including on-going meetings and coordination, stakeholder collaboration and continuous improvement as the program evolves.<br>• **Monitoring and Assessment** of the governance framework including timeliness, responsiveness and adaptiveness to advancing clear decision making and addressing critical issues or challenges
Key Considerations from a Reform Perspective	• **Participatory**: Good governance and as such needs to be informed and organized, with all actors within the system valued and empowered to bring their perspectives.<br>• **Consensus oriented**: Actors within the system will have diverse viewpoints and perspectives. It will be critical to bring voices and the mediation of different interests to drive towards decision making.<br>• **Accountable**: Accountability may carry depending on if decisions are internal or external to the system. Generally, the system is accountable to those who will be affected by decisions or actions- so it is critical to ensure national, sub national and community voices are considered.<br>• **Transparent**: Information must be readily available and accessible to all actors within the system, which will enable effective decision making.<br>• **Responsive:** Good governance requires that institutions and processes try to serve and communicate continuously with all stakeholders within a reasonable timeframe.<br>• **Effective and efficient**: The governance system should produce results and serve its stakeholders by making the best use of resources at their disposal.<br>• **Equitable and inclusive** - A governance system's success will depend on ensuring that all its actors feel that they have a voice and do not feel excluded from providing their perspective into decision making. In this case, this requires considering the voices that may be excluded and having opportunities to include them during key milestones.<br>• **Integrated**: Remember that the community health system is a part of the larger health system. It is a target of and input to health systems governance - ensure that the bi-directional aspect is maintained (e.g., make sure the community health component is properly represented in health sector meetings or annual reviews). All this is in turn part of country governance and interaction with politics, executive agencies, etc.

Resources	● MSH's "Leaders Who Govern" Guide.<br>● USAID Health Finance and Governance project, lessons learned

Management and Learning

During the management and learning stage, actors implement reformed policies and programs and utilize learning and data to inform improved performance of the system. USAID captures this idea in their program cycle as "manage adaptively through continuous learning."[18] Key stakeholders identify gaps in implementation and jointly problem solve to improve performance. These learnings inform immediate improvements, as well as identify larger changes to program design or the community health systems that constrain impact.

Cycle Stage	Management and Learning
Milestones	● Key stakeholders regularly reviewing data to inform joint problem-solving (e.g., regular program reviews at national and subnational level)<br>● Identify and institutionalize reflection points<br>● Continuous improvement within existing program design<br>● Challenges and changes to program design are identified<br>● Other systems bottlenecks are also identified
Key Considerations from a Reform Perspective	● **Adaptive Learning:** Establish quarterly or routine milestones at which key actors responsible for program rollout come together to review program progress, address challenges, assess impact and

[18] USAID. Discussion Note: Adaptive Management, Program Cycle. *USAID;* 2018. Available at: https://usaidlearninglab.org/library/discussion-note-adaptive-management.

	quality. New tools are created and rolled out, responsive to implementation, design and management needs for health systems leaders and actors<br>● **Systems Perspective**: As new policies or programs roll-out, they often reveal other challenges or opportunities within the community health system. Example: a new mHealth payment mechanisms reveals the need to strengthen workforce registration. Consider this as good data on the overall function of the community health system, and signals about where reform may be focused next<br>● **Building the right Evidence**: Capture, articulate, and package evidence to (1) inform program improvements and (2) buy-in for further sustainability and reform. Note that evidence can be quantitative, qualitative, case studies, formal, or informal - so long as it's perceived as effective. Do not neglect the power of "first-hand, experiential evidence... [that fosters] emotional buy-in."[19]<br>● **Performance Monitoring:** Data and learning is generated frequently and is being shared externally to demonstrate progress of program rollout and activities<br>● **Performance & Impact Evaluation:** Research and evaluation agendas are established in which data, learning and assessment of impact can be generated- critical to ensuring continued investment and support from key stakeholders<br>● **Adaptive Design:** As the program/reform grows consider how the design should evolve - especially if translating from small-scale success to larger adoption. For example, considerations for simplification of design, bundling with other complementary initiatives, integration and de-duplication across health sector, or converting from "project" management into a routine service.[20]<br>● **Expect change**: Policy contexts are unlikely to be static. External shocks (e.g. COVID-19), new government priorities, or sometimes even new governments often change the policy and political environment of the reform, sometimes creating new opportunities.<br>● **Continuity of knowledge**: Many donor-funded reform efforts fail when project-funding runs out - prepare early on for how to transfer the knowhow and institutionalize knowledge. Better yet, don't design reform solely around project based-funding.

[19] Spicer N, Hamza YA, Berhanu D, et al. "The development sector is a graveyard of pilot projects!" Six critical actions for externally funded implementers to foster scale-up of maternal and newborn health innovations in low and middle-income countries. *Global Health*. Published online 2018. doi:10.1186/s12992-018-0389-y

[20] Kohl and Foy. Guide to the Agricultural Scalability Assessment Tool for Assessing and Improving the Scaling Potential of Agricultural Technologies. *USAID*; 2018. Available at: https://reliefweb.int/report/world/guide-agricultural-scalability-assessment-tool-assessing-and-improving-scaling

	• **Prepare for Reform Again**: At some point, the context of programming will have changed. Incremental improvements and performance challenges will reveal themselves as new problems to be prioritized. Indeed, community health reforms that have successfully endured evolve and adapt overtime.[21]
Resources	• USAID Discussion Note on Adaptive Management<br>• ODI Supporting Adaptive Management - M&E Tools and Approaches<br>• CHIC - CHW Program Functionality Matrix

[i] Hummelbrunner R, Jones H. A guide for planning and strategy development in the face of complexity. Overseas Development Institute; 2013. Accessed January 25, 2021. https://www.odi.org/sites/odi.org.uk/files/odi-assets/publications-opinion-files/8287.pdf

[ii] Ramalingam B, Laric M, Primrose J. From best practice to best fit: understanding and navigating wicked problems in international development. Overseas Development Institute; 2014. Accessed January 25, 2021. https://www.odi.org/sites/odi.org.uk/files/odi-assets/publications-opinion-files/9159.pdf

[iii] Mansoor Z, Williams MJ. Systems approaches to public service delivery: lessons from health, education, and infrastructure. Published May 7, 2018. Accessed January 25, 2021. https://www.bsg.ox.ac.uk/sites/default/files/2018-06/Background%20Paper-Systems%20Approaches%20to%20Public%20Service%20Delivery%2014-15%20May%202018.pdf

[iv] Senge P, Hamilton H, Kania J. The dawn of system leadership. *Stanford Social Innovation Review*. 2015. Accessed January 25, 2021. https://ssir.org/articles/entry/the_dawn_of_system_leadership

[v] Faustino J, Booth D. *Development Entrepreneurship: How Donors and Leaders Can Foster Institutional Change.* Overseas Development Institute; 2014. Accessed January 25, 2021. https://www.odi.org/sites/odi.org.uk/files/odi-assets/publications-opinion-files/9384.pdf

[vi] Kleinfeld R. In Development Work, Plan for Sailboats, Not Trains. *Stanford Social Innovation Review*. December 2, 2015. Accessed January 25, 2021. https://carnegieendowment.org/2015/12/02/in-development-work-plan-for-sailboats-not-trains-pub-62177

[21] https://www.exemplars.health/.
